# Collection and Analysis of High Temperature‐Resolution ^57^Fe Mössbauer Spectroscopy for Detailed Iron Mineral Characterisation

**DOI:** 10.1002/cphc.70557

**Published:** 2026-09-23

**Authors:** Andrew R. C. Grigg, James M. Byrne, Ruben Kretzschmar

**Affiliations:** ^1^ Institute of Biogeochemistry and Pollutant Dynamics ETH Zurich Zurich Switzerland; ^2^ School of Earth Sciences University of Bristol Bristol UK

**Keywords:** blocking temperature, crystallinity, ferrihydrite, Mössbauer effect, temperature‐dependent fitting

## Abstract

^57^Fe Mössbauer spectroscopy is a powerful technique for determining the oxidation state and magnetic properties of Fe‐bearing materials. Since magnetic properties are temperature dependent, Mössbauer spectra are often collected at multiple temperatures, especially around characteristic transition points such as the magnetic blocking temperature. Mössbauer data acquisition times are slow, sometimes taking >24 h to achieve high signal‐to‐noise ratios (SNR), which limits the number of spectra that can be collected. Here we propose an alternative approach, measuring profiles with high temperature resolution (e.g. 1 K temperature steps) but lower SNR. Using ferrihydrite with varying crystallinity as examples, we revealed mineralogical properties with increased detail even without large increases in the total acquisition time. The temperature dependence of the hyperfine parameters was clearly revealed in qualitative analysis, while simultaneous quantitative analysis of the spectra in both the temperature and energy domains revealed details about the distribution of particle crystallinity in pure ferrihydrite and mixed‐phase samples. This approach will improve the measurement of temperature‐dependent mineral properties, enabling analysis of blocking temperature distributions, improving the interpolation of hyperfine parameters in temperature profiles and aiding the discrimination of overlapping spectra in mixed‐phase samples.

## Graphical abstract

^57^Fe Mössbauer spectra measured at several temperatures can reveal the magnetic properties of Fe‐bearing phases. However, the number of measurement temperatures is limited by long integration times. We present a novel approach to collect high temperature‐resolution profiles, using the temperature relationship of the hyperfine parameters to compensate for a lower signal‐to‐noise ratio and to fit temperature‐dependent properties of ferrihydrite.

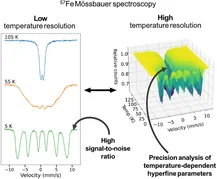

## 1 Introduction

Mössbauer spectroscopy is a powerful analytical technique for physical and chemical sciences based on recoilless emission and resonant absorption of gamma radiation by Mössbauer‐active isotopes. Mössbauer spectroscopy can precisely analyse the electronic state, oxidation state and atomic interactions of target isotopes [1]. The most researched of the Mössbauer‐active isotopes is ^57^Fe, which has been applied to research questions in a range of disciplines dominated by materials science, condensed matter physics, applied physics and chemistry, with significant growth in fields such as chemical engineering and Earth and planetary science [2].

One application of ^57^Fe Mössbauer spectroscopy is the determination of the magnetic properties of Fe‐bearing materials, which arise from the electronic spin configurations of iron ions. These properties are strongly temperature‐dependent, as thermal energy progressively disrupts magnetic ordering. At elevated temperature, the net magnetic field is averaged to zero, and doublet or singlet features are produced in Mössbauer spectra. At lower temperatures, sextets are observed, yielding six or eight resonance lines, depending on the angle between the internal magnetic field vector and the principal axis of the electric field gradient [3]. The transition between these states is observed at the blocking temperature (*T*_B_), where the relaxation time of half of the Fe in the target phase exceeds the characteristic time of the measurement [4]. The transition is operationally defined as the temperature at which half of the spectrum presents as a sextet [5]. The *T*_B_ is typically lower than the material magnetic ordering temperature, which determines whether a mineral exhibits ferromagnetic, ferrimagnetic, antiferromagnetic or paramagnetic behaviour (e.g. Néel temperature for antiferromagnetic minerals such as ferrihydrite). Since *T*_B_ is dependent on the shape and size of the magnetic domains, it is a useful parameter to analyse the crystallinity of a material. Furthermore, analyses of samples containing a mixture of phases above and below the *T*_B_ of each phase can be used to discriminate phases that have similar hyperfine parameters or overlapping spectra that cannot be accurately fit together [3].

Determination of *T*_B_ using Mössbauer spectroscopy typically requires the collection of multiple spectra above and below *T*_B_. For mineral phases with a substantial particle size distribution, spectra measured near *T*_B_ contain mixtures of collapsed sextets and doublets [6]. Therefore, *T*_B_ can be interpolated from the fraction of ordered and unordered sites across a temperature range [3]. Interpolation of *T*_B_ may be linear, although some methods fit non‐linear distributions [5, 7] or approximate *T*_B_ from a single spectrum by applying assumptions about the distribution of *T*_B_ [5]. Nonetheless, in most practical cases, the current approaches do not allow for precise analysis of the average *T*_B,_ or its distribution.

This work introduces an approach to time‐effectively analyse Mössbauer temperature profiles at high temperature resolution (i.e. spectral measurements across a range of temperatures with finely spaced temperature intervals) using the temperature relationship of hyperfine parameters to compensate for lower signal‐to‐noise ratio measurements in qualitative and quantitative analysis at all temperatures simultaneously. The high temperature‐resolution measurements offer more detailed information about temperature sensitivity of the physical parameters of the Mössbauer‐active phases. We introduce the approach with a case study using ^57^Fe Mössbauer spectroscopy of synthetic ferrihydrite minerals with contrasting magnetic ordering properties, and demonstrate how high temperature‐resolution analysis will create new analytical opportunities for Mössbauer spectroscopists in diverse fields of research.

## 2 Experimental Section

Samples of the mineral ferrihydrite with varying crystallinity were synthesised by neutralisation of ^57^Fe(III) solutions with controlled additions of KOH [8]. 6‐line ferrihydrite (6L‐Fh) was produced by partially neutralising the Fe(III) solution to pH 2 with 800 mM and 200 mM KOH, heating to 80 °C, cooling to room temperature and raising to pH 7 with 200 mM KOH at a rate of 1 mL/min. Two‐line ferrihydrite (2L‐Fh) was synthesised by dropwise addition of 800 mM KOH to an Fe(III) solution at a rate of 1 mL/min. Low crystallinity 2‐line ferrihydrite (2Lc‐Fh) was produced by suspending 10 g/L soil in the Fe(III) solution during the 2‐line ferrihydrite synthesis procedure. Mineral precipitates were rinsed and freeze dried, before their purity was demonstrated by X‐ray diffraction (Figure S1). Dried mineral samples were sealed in Kapton tape for Mössbauer measurements. Full details of mineral preparation are available in Section S1.1.

Mössbauer measurements were carried out using a WSS‐10 spectrometer (WissEL, Germany) with ^57^Co/Rh γ‐radiation source, 7 mm thick α‐Fe(0) foil for calibration and closed‐cycle liquid‐He cryostat (SHI‐850, Janis Research, USA) with Lakeshore 335 cryostat controller for measurements below room temperature. Spectra were collected through the WissEL proprietary software, under the automated control of custom‐written python code [9]. The automation allowed real‐time analysis of spectral quality via calculation of the signal‐to‐noise ratio (SNR). SNR was calculated by comparing the strongest resonance peak with the standard deviation of the noise on both ends of the spectrum [10, 11], normalised by the spectral area. Once predefined quality targets were met, the measurement temperature was lowered and data collection reset. Details of the automation procedure are available in Section S1.2.

Individual temperature spectra were fitted using an Extended Voigt‐Based Fitting (xVBF) line shape [12]. High temperature‐resolution datasets were displayed as velocity–temperature matrices for qualitative interpretation. Quantitative analysis applied three fitting protocols, based on an xVBF line shape [12], an xVBF line shape with modified Lorentzian linewidth for the four inner sextet lines proportional to spectral relaxation time, *τ* [13] and a dynamic relaxation model based on a stochastic two‐phase model of spin flipping [14] as implemented in SYNCmoss software (Section S1.3) [15]. To simultaneously fit all spectra in the temperature profile, the temperature‐dependency of the hyperfine parameters were calculated from a fitted value at saturation (0 K). Above 0 K, centre shift (*δ*) was calculated according to its fitted value at 0 K, the temperature‐independent isomer shift and second‐order doppler shift [1]. Hyperfine field was calculated by the Brillouin function [16] with magnetic field strength calculated according to Bean and Rodbell [17]. Spectral area was normalised by the Lamb–Mössbauer factor [18]. Fitting was undertaken using the ‘Scipy optimize’ (Scipy v1.16.3) package [19]. Full details of the fitting methodology and modelling of temperature‐dependent parameters are available in Section S1.3 and the published code [9].

## 3 Results and Discussion

**FIGURE 1:**
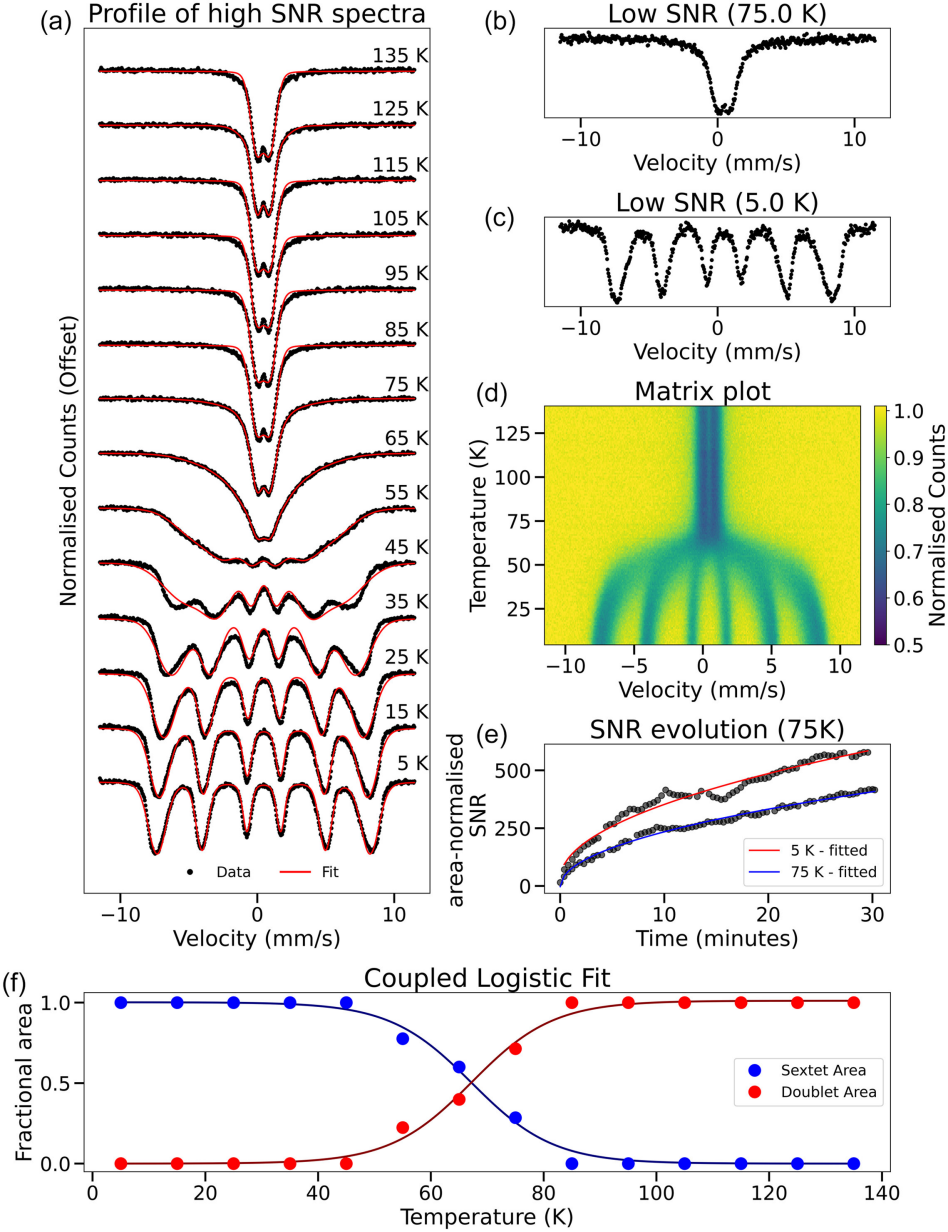
Comparison of a high‐ and low‐resolution temperature profile of ^57^Fe‐enriched 2L‐Fh, each with the same total collection time. (a) High signal‐to‐noise (SNR) spectra with 30 min collection time for each at 10 K resolution (fitting parameters in Table S1). (b,c) Two example low‐SNR spectra with 3 min collection time. (d) All low‐SNR spectra from a high temperature‐resolution profile plotted in a matrix of energy and temperature. (e) Evolution of the area‐normalised SNR during the measurement of high SNR spectra. (f) Logistic function fits of the fraction of doublets and sextets in spectra from panel (a).

The analysis of Fe‐bearing materials by Mössbauer spectroscopy involves measurements at several temperatures. To measure a Mössbauer temperature profile, the distribution of measuring temperatures is carefully chosen according to the properties of the sample and the requirements for effective data analysis. For example, the temperature resolution of a Mössbauer profile affects the measurement precision of key mineral properties such as the *T*_B_. To illustrate this effect, a low temperature‐resolution profile of ^57^Fe‐enriched 2‐line ferrihydrite (2L‐Fh, Section S1.1) was measured at 10 K intervals for 30 min at each temperature (Figure 1a). To estimate *T*_B_, relative areas corresponding to sextets and doublets were fitted at each temperature. Up to two doublets and one sextet based on an xVBF line shape were needed to effectively model the full area (Section S1.3). Changes in the areas with temperature were fitted using a logistic curve (Figure 1f) [7].

The 10 K resolution was sufficient to estimate *T*_B_ to within several K in this example, because the spread of *T*_B_ was large and 10 K is a relatively high resolution for a standard temperature profile. Precision falls when too few spectra contain a mixture of doublet and sextet to effectively constrain the gradient of the logistic curve. Importantly, the details of the fitting protocol at each temperature can have profound impacts on the estimation of *T*_B_. This is because low temperature‐resolution data only have a small number of spectra containing a mixture of sextets and doublet and because of the difficultly of unambiguously assigning the correct area to sextets and doublets in spectra with a mixture of doublets and partially ordered sextets with minimal context from spectra measured at neighbouring temperatures.

In response to the limitations of low temperature‐resolution Mössbauer spectral characterisation of Fe‐bearing materials, we have developed a method to time‐effectively analyse Mössbauer temperature profiles at high temperature resolution. This is possible because most spectral information is gained at the beginning of a measurement. According to the scaling law for the signal‐to‐noise ratio (SNR) of a measurement variable that is subject to random noise, the increase of the SNR is proportional to $\sqrt{N}$ where *N* is the number of measurement cycles (Figure 1e) [20]. Therefore, the marginal benefit of doubling the count time is a 41% improvement in the SNR, and a ten‐fold increase in the count time is a 216% improvement in the SNR. We collect spectra to lower SNR during the most efficient period of the measurement, while using the saved counting time to increase the temperature resolution of the analyses. For example, a high temperature‐resolution profile of ^57^Fe‐enriched 2L‐Fh was measured at 1 K intervals and plotted in the energy and temperature domains simultaneously (Figure 1c) with a colour scale to indicate the relative absorption intensity (Section S1.2). Each temperature was measured for 3 min, therefore taking the same total acquisition time as the low temperature‐resolution profile (Figure 1a). Since changes to the spectral shape with temperature are incremental, the effect of spectral noise can be compensated by comparison of spectra at adjacent temperatures, either visually (qualitative analysis) or by fitting known temperature dependencies of hyperfine parameters (quantitative analysis).

**FIGURE 2:**
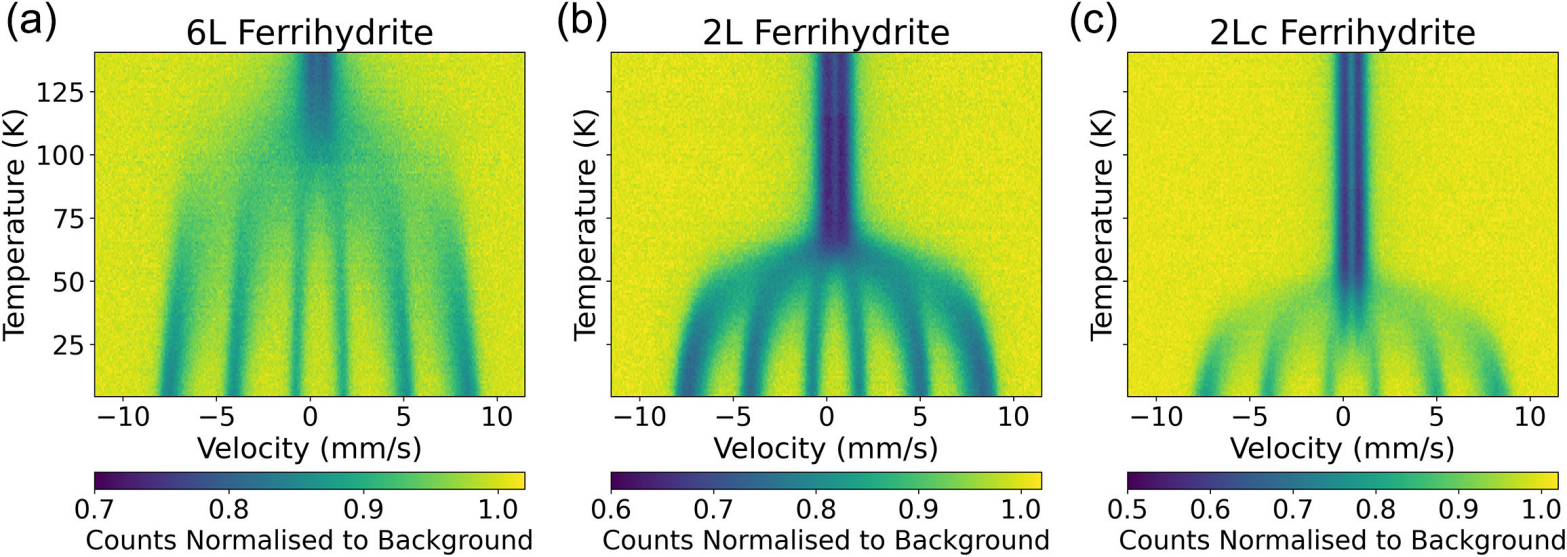
Plots of high temperature‐resolution data for three samples of ferrihydrite. (a) 2L‐Fh (same sample as in Figure 1), (b) 2Lc‐Fh and (c) 6L‐Fh.

For qualitative analysis, high‐resolution temperature profiles facilitate direct visual comparison of the magnetic properties of several samples and improve the communication of Mössbauer spectral interpretations. For example, we examined three synthetic ferrihydrite mineral samples: 6‐line (6L‐Fh), 2‐line (2L‐Fh) and 2‐line ferrihydrite coprecipitated with soil (2Lc‐Fh) (Section S1.1). The differences in *T*_B_ are evident when comparing their respective Mössbauer spectra at 55 K (Figure S5). However, the high temperature‐resolution data provide a clearer characterisation including the spread of *T*_B_ among particles in the sample. Figure 2 demonstrates that 6L‐Fh has a high *T*_B_, consistent with higher crystallinity [21], and large *T*_B_ distribution, consistent with a large range of particle sizes [5]. In comparison, the 2Lc‐Fh doublet persists to lower temperatures than the main region of magnetic ordering around 40 K, which could suggest that the distribution of particle crystallinity is not Gaussian or that multiple ferrihydrite phases with contrasting crystallinities formed. The irregular distribution could be caused by the interaction of a fraction of the particles with soil components during crystal formation.

**FIGURE 3:**
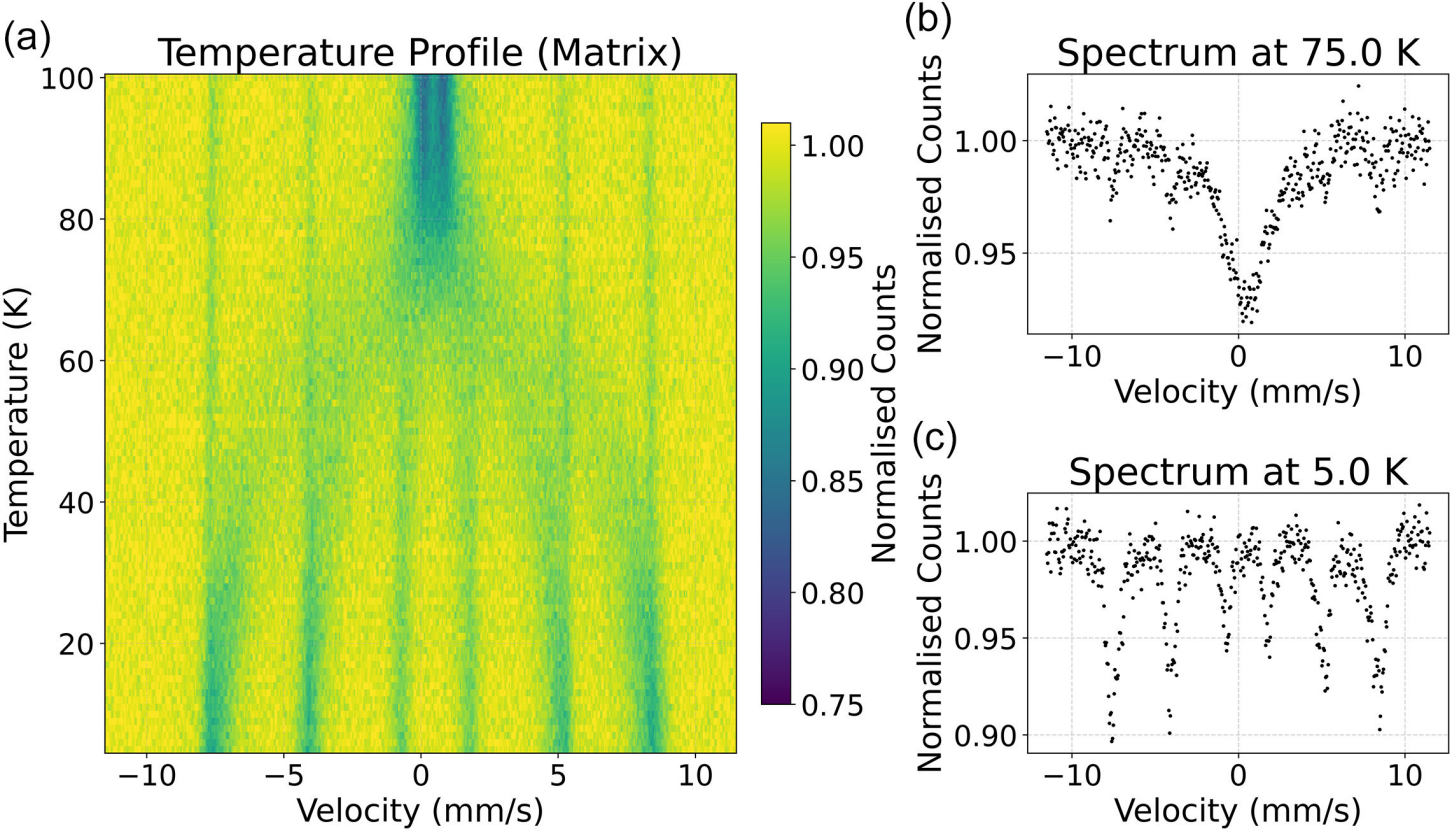
A mixture of 16% ^57^Fe‐enriched goethite and 84% ^57^Fe‐enriched ferrihydrite was measured at high temperature resolution, demonstrating how the profiles can be used to discriminate different phases in a sample, and to demonstrate the presence of low‐abundance phases. (a) The full dataset is plotted as a matrix with (b,c) two subsets presented as single spectra illustrating the level of noise inherent in these measurements.

The high temperature‐resolution profiles offer detailed information about temperature sensitivity of the physical parameters of the Mössbauer‐active phases. This may be used to reveal the presence of multiple phases with contrasting average *T*_B_ or *T*_B_ distributions (Figure 3a). Since it may not be possible to identify or accurately fit overlapping phases in spectra from single measurement temperatures (Figure 3c), observation of several *T*_B_ values may help to reveal how many phases are present and thereby inform the analysis of single spectra. Image processing techniques may also be used to automatically identify spectral features (Figure S6).

Quantitative analysis of temperature‐dependent parameters is also possible from high temperature‐resolution data. Mössbauer spectral parameters at all temperatures are fundamentally related to one another and high‐resolution temperature profiles offer unprecedented detail for the fitting of spectral parameters across temperatures. The potential benefits include not only fitting of hyperfine parameters in individual temperature spectra within the full context of the profile, but also the quantification of temperature‐dependent parameters.

**FIGURE 4:**
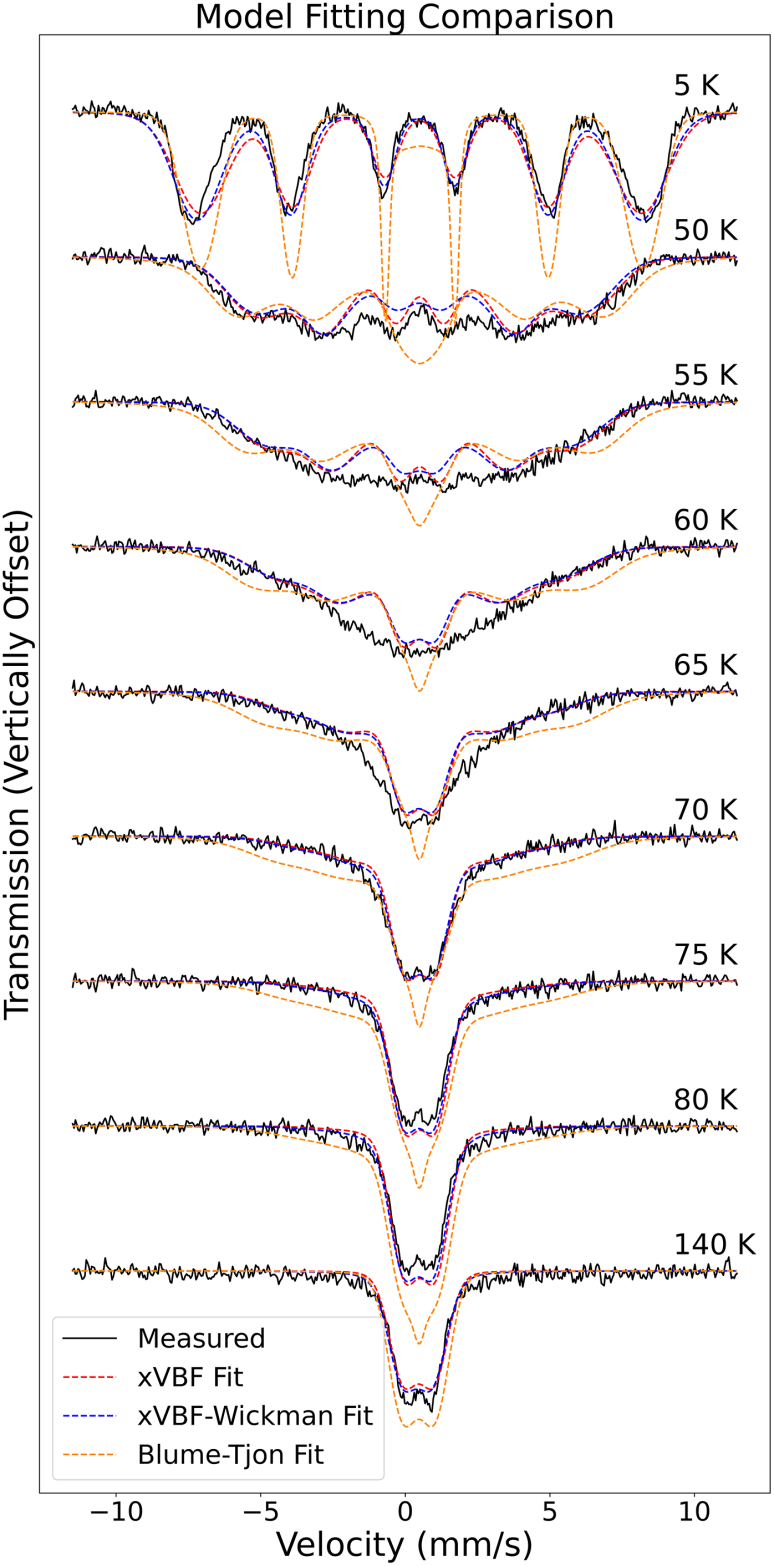
The fitting of the spectra is dependent on the chosen fitting model. Selected spectra are shown from the high temperature‐resolution profile of 2L‐Fh and overlaid by fitted spectra, based on parameters from three fitting protocols, applied to all 135 temperatures simultaneously. All fitted spectra are shown in matrix format in Figure S7.

We applied three spectral line profiles to fit 2L‐Fh data in two dimensions over a temperature range of 5–140 K and velocity range of −12 to +12 mm/s (Figure 4). Firstly, we applied xVBF [12] as an example of a static relaxation model (Section S1.3). The advantage of xVBF is that it can fit distributions of all hyperfine parameters. However, it is not designed to model spectral line shapes near *T*_B_ where dynamic relaxation and the magnetic relaxation time (*τ*) influence the spectral form. Therefore, we also applied a modification of the xVBF line shape with line broadening proportional to *τ* (called xVBF‐Wickman in Figure 4, details in Section S1.3) [13]. Finally, to test a dynamic relaxation model, we applied the Blume–Tjon two‐phase probabilistic model of spin‐flipping [14]. While the Blume–Tjon model is designed to reflect the physical causes of the collapsed line shape, it is unable to model static broadening of hyperfine parameters, which we mimicked by applying Gaussian smearing to the spectrum in six sections.

Since the fits are undertaken at all temperatures simultaneously, a key aspect of the fitting protocols is the relationship between temperature and fitting parameters. This includes the effect of distributions in *T*_B_ on spectral shape at each temperature. Therefore, in applying all three protocols, the line shape at each temperature is derived from the superposition of several spectra with hyperfine parameters that vary according to the distribution of *T*_B_ (Figure S9). This models the asymmetrical broadening of peaks observed in many partially ordered sextets, provides a first‐order approximation of the collapsed feature using static relaxation models, and improves the line shape produced by the dynamic relaxation model near *T*_B_. The large distributions of *T*_B_ (9–12 K 1SD, Table 1) in the 2L‐Fh highlights the potential impact of this parameter for the line shape of energy‐domain spectra around *T*_B_.

**TABLE 1:** Fitting parameters for the 2L‐Fh using three contrasting fitting models, with comparison to static fitting of high‐SNR spectra. The Debye temperature (*θ*_D_) was pre‐fit to the measured area of the spectra, which informed the initial guess of 110 K.

	xVBF	xVBF–Wickman	Blume–Tjon	Static fit **(**Figure 1 **)**
*θ*_D_, K	155.80	167.83	117.05	N/A
*δ*_0_, mm/s	0.58	0.53	0.58	N/A
QS_0_, mm/s	1.07	1.05	1.07	N/A
<|*B*_sat_|>, T	47.82	48.18	47.39	N/A
*T*_B_, K	64.23	62.97	78.61	67.25
*σ* (QS), mm/s	0.90	0.85	0.88	See Table S1
*σ* (*ε*), mm/s	0.37	0.29	0.46	See Table S1
*σ* (<|*B*_hf_|>), T	2.70	2.50	3.35	See Table S1
*σ* (*T*_B_), mm/s	9.41	10.92	11.67	N/A
*σ *(*T*_B_) step resolution	100^a^	100^a^	20^a^	N/A
*ν*	0.30	0.23	0.24	N/A
*θ*, rad	0.93	0.96	0.92	N/A
Intensity	5.84	5.60	4.38	N/A
log_10_(f_0_)	N/A	12.45	8.40	N/A
*A*	N/A	0.01	N/A	N/A
*B*	N/A	0.05	N/A	N/A
*C*	N/A	0.02	N/A	N/A
Г, mm/s	0.135^a^	0.135^a^	0.135^a^	0.135^a^
Reduced *χ* ^2^	2.78	2.37	13.58	N/A
Global NRMSE, %	2.70	2.49	5.73	N/A

a Constant parameters that were not floated in the final fitting protocol.

The hyperfine parameters extracted from the 2D fits at key temperatures are comparable to those obtained from Voigt‐based fitting and xVBF fitting in previous studies [22, 23]. Although two‐ and three‐component models have been proposed for ferrihydrite [24], a single xVBF component, especially with fitted variability of the quadrupole shift, provides a reasonable estimation for some applications [22, 23] including the fitting in Figure 1a. Direct comparisons between the temperature profile fits and single‐temperature fits are not appropriate for partially collapsed spectra near *T*_B_, but the misfits of the collapsed features produce errors that affect the results at key reference temperatures such as 5 and 110 K. The results of the static model and Wickman dynamic model are most similar to the fits of high‐SNR spectra at key reference temperatures.

One cause of the variability between the fitting protocols was misfitting of the partially collapsed spectra near *T*_B_ (Figure S8). None of the three models could fully capture the temperature dependency of hyperfine parameters, possibly because they do not account for all of the irregular magnetic behaviour of ferrihydrite [25, 26]. For instance, the fitted *T*_B_ of 2L‐Fh varied substantially for the three fitting protocols (Table 1). The static model produced an estimation of *T*_B_ (64.23 K) closest to the logistic fit of doublet area in individual spectra, with a similar estimation (62.97 K) from the Wickman model. These models produced the lowest *χ* ^2^ (Table 1) and best reproduction of key spectral features at 5 K, 110 K and near *T*_B_ (Figure 4). By contrast, the fit using the Blume–Tjon model for dynamic relaxation could not capture several key features of the spectra, such as the shape of the saturated sextet, breadth of the collapsed sextet, or separation between the doublet peaks close to *T*_B_ (Figure 4). In combination, these examples of misfitting led to an elevated estimation of *T*_B_ by the Blume–Tjon model.

The optimised values of several temperature‐dependent hyperfine parameters are likely conflated with missing parameters in these models. Low fitted values for the temperature dependence of the hyperfine field in all fitting models (*ν*, ~0.3, Table 1) could indicate a soft transition of the B‐field from saturation at 0 K to 0 T at *T*_B_, due to nanoparticle surface effects of ferrihydrite [27]. However, the pattern of misfitting (Figure S8) suggests that the hyperfine field (*B*_hf_) gradient with respect to temperature is underestimated. The fitted value of *ν* is used in the three fitting protocols to capture ill‐defined collapsed features with low *B*_hf_ visible in Figure 4. Similarly, the fitted value of the Debye temperature (*θ*_D_) was lower than that of similar materials such as ferritin [28]. The fitted value is affected by edge effects or recoil of low‐mass nanoparticles [29], but also saturation effects of thick absorbers. Most importantly, it is very sensitive to the temperature‐dependent changes of each of the hyperfine parameters, and a compromise value of *θ*_D_ is fitted to satisfy each of the parameters.

In interpreting the results of the qualitative analysis, there are two key principles to consider. Firstly, the fitted parameters reflect functional properties of the nanoparticles rather than bulk material properties. This can be exploited to understand mineralogical properties such as crystallinity but may mean that the values of each parameter are affected by the conflation of several physical factors. Secondly, whereas fitting of single‐energy Mössbauer spectra often requires the use of physically implausible parameters to produce a reasonable fit, the success of temperature‐based fitting is more reliant on the applicability of the model to the physical characteristics of the sample. Models that do not capture key material properties at all temperatures may produce compromises in fitting outcomes, especially when observed for individual temperatures as in Figure 4. Crucially, these model insufficiencies also impact the fitting of individual temperature spectra, even if the effects are less noticeable. The results of this study demonstrate that further work is required to understand the magnetic blocking behaviour of ferrihydrite.

The high‐resolution temperature profiles require processing of large datasets, and the computational costs of quantitative analysis may be limiting. Qualitative analysis is computationally trivial, and the quantitative static relaxation models were run in less than 2 h each on a dedicated standard workstation (Intel Core i7, 8 cores, 32 GB RAM). However, the dynamic relaxation model required 15 h of computation. Compromises that could reduce computational time include fewer iterations of the fitting model and recursive B function, lower resolution of numerically integrated distribution functions and a smaller temperature range. Quantitative analysis of the profiles will benefit from innovation in fitting protocols, especially modelling of relaxation phenomena and temperature dependency of parameters. The modelling of collapsed features remains the subject of debate [6], and currently the choice of relaxation model depends on the nature of the sample [30]. We anticipate that increasing availability of Mössbauer spectroscopy and computational power will drive innovation in the collection and analysis of Mössbauer temperature profiles.

Given the collection time required for 1 K intervals, the high temperature‐resolution profiles will most often replace low temperature‐resolution profiles with a relatively large number of high‐SNR spectra. For example, in this study, we demonstrate the replacement of 10 K intervals with 1 K intervals using an equivalent total collection time. In practice, the collection time of low‐SNR spectra need not be 10% of the high‐SNR spectral collection time. Instead, collection time should be decided on a case‐by‐case basis, depending on the purpose of the analysis and nature of the sample. In the examples presented in Figures 1 and 3, ^57^Fe enrichment reduced the acquisition time to 3 min for low‐SNR spectra, and 30 min for high‐SNR spectra. However, samples containing natural Fe isotope ratios (the Fe in unenriched samples contains 2.119% ^57^Fe [31]) have also been measured successfully with this protocol employing longer collection times as necessary. Collection times vary widely depending on sample and instrument properties, as well as analysis objectives, but low‐SNR measurements of natural isotope‐abundance Fe minerals typically require at least 30 min per temperature. In every case, users must accept a trade‐off between the SNR required to see low‐abundance features and the acquisition time that may be afforded, as well as the temperature range of the analysis, and potentially the temperature resolution applied. A benefit of the high‐resolution profile is that some phases may be identifiable in the matrix even if the low SNR prevents identification in individual spectra (Figure 3).

The measurement of the high temperature‐resolution profiles requires an automated collection protocol (Section S1.2). The protocol proposed here uses either the SNR or time as a threshold to terminate spectral acquisition at each temperature. SNR‐based thresholds are useful when the required time is difficult to predict a priori, but the required spectral quality is known. Area normalisation of the SNR is applied to reduce the influence of spectral form on SNR (Section S1.2), although differences remain (Figure 1e). Real‐time SNR analysis may also be used to optimise the acquisition time for any single Mössbauer spectrum. The automation protocol (Section S1) was based on discrete temperature steps, but future development of a continuous scanning protocol will eliminate the need to pause data collection while the temperature is adjusted. Details of the algorithm used for automatic collection and temperature‐based fitting, is available in the Supporting Information and published code [9].

This method will have immediate applications in environmental geosciences, including soil science and environmental mineralogy. Crystallinity, including crystallite size, long‐range order and element substitution, controls mineral reactivity in the environment [32] and is commonly assessed using Mössbauer spectroscopy [3]. However, poorly crystalline phases are difficult to identify in single‐temperature Mössbauer spectra because of large hyperfine parameter distributions. Moreover, it can be challenging to choose temperatures that avoid overlapping spectra or partially magnetically ordered phases with large crystallinity distributions in samples containing multiple phases, such as natural samples [33]. Beyond environmental science, this approach is broadly applicable to many types of samples and research disciplines.

## 4 Conclusion

High temperature‐resolution Mössbauer spectroscopy measurements offer a unique insight into the magnetic properties of Fe‐bearing materials, by shifting the trade‐off between SNR and temperature resolution in Mössbauer analysis. Whether analysed qualitatively or quantitatively, the high temperature‐resolution data provides additional clarity on a material’s crystallinity and may reveal low‐abundance phases in complex mineralogically mixed samples. The automated protocol facilitates time‐efficient measurements, offering increased temperature resolution without increased demands on instrument time. As a tool that offers rigorous characterisation of Fe‐bearing materials and mineral mixtures, we anticipate that high temperature‐resolution Mössbauer spectroscopy will provide unique value to projects in a range of scientific disciplines.

## Funding

This research is part of a project that has received funding from the European Research Council (ERC) under the European Union’s Horizon 2020 research and innovation programme (788009‐IRMIDYN‐ERC‐2017‐ADG). J.M.B was supported through a UKRI Future Leaders Fellowship UKRI2857.

## Conflicts of Interest

The authors declare no conflicts of interest.

## Supporting information

Supplementary Material

## Data Availability

The data and code that support the findings of this study are openly available on Github at https://github.com/Andrew‐R‐C‐Grigg/high‐temp‐res‐mossbauer‐profiles and Zenodo at https://doi.org/10.5281/zenodo.21971232.
