# Supplementary material for: Collection and Analysis of High Temperature‐Resolution 57Fe Mössbauer Spectroscopy for Detailed Iron Mineral Characterisation
Source: Chemphyschem. 2026 Sep 23;27(18):e70557. doi: 10.1002/cphc.70557 (PMC13600951; doi:10.1002/cphc.70557)
Supplement: Supplementary file 1 [file CPHC-27-e70557-s001.pdf]

Supplementary information for

Collection and Analysis of High Temperature-Resolution ^{57}Fe Mössbauer Spectroscopy for Detailed Iron Mineral Characterisation

Andrew R. C. Grigg[1], James M. Byrne[2], Ruben Kretzschmar[1]

[1] Institute of Biogeochemistry and Pollutant Dynamics, ETH Zurich, CHN, Universitätstrasse 16, 8092 Zurich, Switzerland

[2] School of Earth Sciences, University of Bristol, Queens Road, BS8 1RJ, Bristol, UK

Table of Contents

S1. Methods 2
S1.1. Mineral synthesis 2
S1.2. Mössbauer data measurement 3
S1.3. Mössbauer data analysis 4
S1.4. Additional supporting analysis: X-ray diffraction 6
S1. Additional Supporting Results 7
S1.1. X-ray diffraction 7
S1.2. Mössbauer Collection Software 8
S1.3. Mössbauer spectral results and data 9
S2. Data Availability 15
S3. References 16

This document contains 17 pages

S1. Methods

S1.1. Mineral synthesis

Ferrihydrite was synthesised by hydrolysis of an Fe(III) solution. For a ferrihydrite sample with high crystallinity and magnetic blocking temperature, 6-line ferrihydrite (6L-Fh) was synthesised. In this procedure, 2.5 mmol ^{57}Fe-enriched Fe(0) powder (96.14 % 57Fe, Isoflex, USA) was dissolved with 0.1 mol HNO_3 (NORMATOM, VWR) diluted to 8.4 M, with constant stirring using a magnetic stir bar, and then diluted to 100 mL by slow addition of KOH solution (50 mL of 800 mM KOH, then 40.5 mL of 200 mM KOH). The Fe(II) in the resultant solution was oxidised by addition of excess H_2O_2 (1.5 mL of 35% H_2O_2, Merck), allowed to react for an hour, and passed through a 0.22 µm nylon syringe filter (BGB, Switzerland). The solution was then adjusted to pH 2.0 by further additions of KOH, taking care to avoid formation of precipitates. The resulting golden-yellow solution, with volume of 170 mL, was heated to 75 °C for 20 minutes to form a deep red colour, cooled at room temperature for 15 minutes, and cooled in an ice bath for a further 15 minutes.[1] The solution was neutralised by the addition of 200 mM KOH solution at a rate of 1 mL/min using an automatic titrator. The precipitates were repeatedly rinsed with UPW, centrifuged (2000 rpm, 1070g, 10 mins) and decanted until the solution conductivity was below 200 µS cm^{-1}.

To synthesise 2-line ferrihydrite (2L-Fh), 5 mmol ^{57}Fe-enriched Fe(0) powder (96.14 % ^{57}Fe, Isoflex, USA) was dissolved with 0.15 mol HNO_3 (NORMATOM, VWR) with constant stirring using a magnetic stir bar, and then diluted to 50 mL (final acid concentration of 3 M without losses due to reaction with Fe). The Fe(II) in the resultant solution was oxidised by addition of excess H_2O_2 (1.5 mL of 35% H_2O_2, Merck), allowed to react for an hour, and passed through a 0.22 µm nylon syringe filter (BGB, Switzerland). The solution was neutralised by the addition of 800 mM KOH solution (made from analytical grade pellets, WVR) at a rate of 1 mL/min using an automatic titrator[2]. The precipitates were repeatedly rinsed with ultra-pure water (UPW, >18MΩ cm, Puranity, VWR), centrifuged (2000 rpm, 1070g, 10 mins) and decanted until the solution conductivity was below 200 µS cm^{-1}.

To produce a ferrihydrite sample with lower crystallinity than the 2L-Fh, 2-line ferrihydrite-soil coprecipitates (2Lc-Fh) were synthesised. In this procedure, 2.5 mmol ^{57}Fe-enriched Fe(0) powder (96.14 % 57Fe, Isoflex, USA) was dissolved with 0.1 mol HNO_3 (NORMATOM, VWR) at a concentration of 8.4 M, with constant stirring using a magnetic stir bar, and then diluted to 50 mL with 800 mM KOH. The Fe(II) in the resulting solution was oxidised by addition of excess H_2O_2 (1.5 mL of 35% H_2O_2, Merck) and allowed to react for an hour with constant stirring. Separately, a forest topsoil from Switzerland (oven dry and sieved to <2 mm) was suspended in UPW at a concentration of 10 g/L, adjusted to pH 3 with HNO_3 and allowed to equilibrate for 48 hours. The Fe solution was added to the soil suspension dropwise by passing the solution through a 0.22 µm nylon syringe filter (BGB, Switzerland). The suspension was neutralised by the addition of 800 mM KOH solution at a rate of 1 mL/min using an automatic titrator with constant mixing by a magnetic stirring bar. The precipitates were repeatedly rinsed with UPW, centrifuged (2000 rpm, 1070g, 10 mins) and decanted until the solution conductivity was below 200 µS cm^{-1}.

After the rinsing, all mineral suspensions were flash frozen by dropwise addition to liquid nitrogen, freeze dried and lightly ground in an agate mortar, to produce poorly aggregated ferrihydrite [3]. All minerals were stored in amber glass vials in a desiccator until used. X-ray diffraction analysis (Figure S1) confirmed the identity of the 2L-Fh and 6L-Fh. The XRD pattern of 2Lc-FH was not measured, due to the difficultly of identifying small amounts of ferrihydrite among the other crystalline soil minerals present.

S1.2. Mössbauer data measurement

Mössbauer spectroscopy measurements were undertaken on the synthetic minerals. Mineral samples for Mössbauer analysis were sealed in polyimide film tape (Kapton tape, 3M Company, USA). Spectra were measured on a WSS-10 spectrometer (WissEL GmbH, Germany) in transmission using a $^{57}Co/Rh$ γ-radiation source. Calibration of the velocity scale was carried out by measurement of a 7 μm thick α-Fe foil at room temperature. For measurements taken below 295 K, a closed-cycle liquid-He cryostat (SHI-850, Janis Research, USA) was used to cool the samples during the measurement.

Measurements were collected using the WissEL proprietary software. The operation of the WissEL software was automated using a software package built around the *pywinauto* python package[4]. The automation program saves progress spectra at user-defined intervals. At each refresh interval, the spectra are saved to the hard-drive, folded with a user-defined calibration file, and then plotted in the accompanying graphical user interface (GUI) to improve the spectroscopist's judgement of the progress of each measurement. In addition, the signal to noise ratio (SNR) or area-adjusted SNR (aSNR) is plotted. The SNR is typically defined as the ratio of the size of the largest resonance peak to the standard deviation of the noise [5]. However, the resonance depth of a doublet is much larger than a sextet, even if the same spectral area is involved. Therefore, area normalisation was applied to reduce the effect of the form of the spectrum on the measure SNR. In the automated fitting program, the SNR or aSNR is fitted with the function $R = k\sqrt{t} + a$ where R is the SNR or aSNR, k is an empirical proportion that is sample specific, t is the acquisition time and a is a factor that take into account that the SNR of a spectrum without signal is not zero because of the effect of the noise itself. On the basis of that calculation, a predicted measurement time is marked on the plot of measured and predicted aSNR in the top right corner of the spectrum collection GUI (Figure S2).

To automate the temperature change, each saved spectrum is analysed for the aSNR and compared to a user-defined aSNR threshold. Alternatively, a time-based threshold can be programmed. Once the threshold is met, the final spectrum is saved, and the temperature in the cryostat is reduced by a pre-defined interval, which was 1 K in all examples presented in this paper. The next measurement takes place when the temperature is within 0.05 K of the setpoint temperature. Command of the Lake Shore Cryogenics (USA) temperature controller is made via the Lake Shore python package called 'lakeshore'[6]. The cryostat was run under vacuum above 110 K and filled with He for faster temperature changes once the temperature dropped below 110 K. Otherwise, the He level in the chamber was left unchanged during the measurement of the profile. The automation protocol is currently based on discrete temperature steps, but future development of a continuous scanning protocol will eliminate the need to pause data collection while the temperature is adjusted.

The code for automated spectral collection is available on Github and through the Zenodo repository [7]. Along with the version used for WissEL software and model 335 cryostat controller, an alternative for SEE Co spectrometers paired with Model 336 temperature controller is also available. Change to the codes for individual system requirements are likely to be necessary, because the code is not currently designed to interface with the large variety of software and hardware in use for Mössbauer spectroscopy measurements. Python packages are available from Lake Shore to interface with other models of controllers that they manufacture. Additionally, *Pywinauto* can be customised to operate any GUI and can therefore be adapted to any GUI interface used to operate Mössbauer spectrometers.

S1.3. Mössbauer data analysis

For qualitative analysis, spectra from high temperature-resolution profiles may be plotted in the energy and temperature domains simultaneously using a matrix approach. In Figure 1, 2 and 4, the profiles are presented as a matrix with energy on the x axis and temperature on the y axis. All spectra are normalised by dividing each datapoint by a background value, determined as the average of the 30 points on either end of the spectrum. This way, the relative areas are preserved. The number of points may be varied depending on the width of the hyperfine field of minerals expected in the sample. The relative transmission is plotted with a colour scale, with the saturation of the colour scale chosen to closely bracket the range of measured values.

Qualitative analysis may be enhanced using image processing tools to find coherent features in the 2D datasets. In this work, we have applied a Steger algorithm, which uses the eigenvalues of a Hessian matrix to identify curvilinear structures in the data. This was implemented using the 'Multi-scale Ridge Detector' python package[8], with pre-application of a gaussian smoothing kernel in two dimensions.

For qualitative analysis, each spectrum in the low temperature-resolution profile (Figure 1) was fit using an extended Voigt-based fitting model (xVBF) [9], without coupling the fit between temperatures. Each spectrum was fit with two doublets and a sextet with the same initial guess parameters using an automated protocol with the fitting engine from the 'Scipy optimize' package[10]. Two doublets were used in the fit due to the broadening of the base of the ferrihydrite doublet, which cannot be accurately fit using a single doublet feature. Such broadening is characteristic of short-range ordered minerals, which can exhibit variations in Mössbauer parameters that are not captured by simple distributions of fitted hyperfine fields. The misfit using a single doublet led to implausibly high estimations of the sextet fraction, and therefore interference in the fitting of the logistic function to the fraction of the sextet in each spectrum. The reported mean and standard deviation of the hyperfine field (B_{hf}) was also reported as an adjusted value, $<|B_{hf}|>$, which ensured that all B_{hf} values in the distribution were positive, by folding the gaussian distribution.

For qualitative analysis of high temperature-resolution profiles, three fitting models were developed. First, a static relaxation model was applied, using the extended Voigt-based fitting model (xVBF) [9] that was originally developed for Recoil (University of Ottawa, 1998). The principles were adapted into the python language, similar to the implementation for Minsight software[11]. The fundamental parameters that describe the xVBF model are centre shift (**δ**), quadrupole shift (QS, for doublets), distribution of the quadrupole shift (σQS, modelled as a gaussian), quadrupole splitting (ε), distribution of the quadrupole splitting ($\sigma\varepsilon$, modelled as a gaussian), B_{hf} and distribution of the hyperfine field (σB_{hf}, modelled as a gaussian). The width of the Lorentzian portion of the peaks was fixed at $\Gamma = 0.135$ mm/s, the inner line broadening of an α-Fe calibration foil measured at room temperature on the same instrument. Additional parameters were also used to describe the temperature dependence of the spectra to the xVBF parameters, namely the blocking temperature (T_B) and distribution of the blocking temperature ($\sigma(T_B)$) for implementation of a gaussian model for the distribution), the Debye temperature (θ_D), and the temperature dependence of the hyperfine field (ν).

The xVBF model uses convolutions of gaussian and Lorentzian line shapes for each of the hyperfine parameters, δ (not implemented in this study), ε and H, to model variability of these parameters between particles in a sample[9]. To extend this application to take account of the effect of the distribution of T_B, a numerical convolution was used for spectra calculated with an assumed distribution of the hyperfine field around T_B. All spectra were modelled as the addition of a doublet and a compound sextet component in a ratio that was defined by the σT_B, with visible consequences for the curve shape near the T_B. Although this produced additional broadness of the peaks around the T_B, it was not sufficiently

broad to match the collapsed line shape of the function around that temperature. Therefore, additional models were sought to model the broadening of the collapsed feature.

As a second fitting model, a modified version of the xVBF model was also implemented, based on the Wickman method of simulating collapsed features[12]. This method relies on the basis of the xVBF model, but ties the broadness, Γ, of the inner four lines of the sextet to the relaxation time of the spectra, W_{relax}. By defining T_B as the temperature at which the Mössbauer measurement time is the same as the relaxation time, τ, we define the energy barrier for spin flipping as [13]:

$$E = k_B T_B \ln(f_0 \tau)$$

with k_B being the Boltzman constant and f_0 being a fitted factor known as the attempt frequency of the magnetic moment flipping. Therefore, the Arrhenius equation is:

$$W_{relax} = K f_0 \exp\left(\frac{-T_B\ log(f_0 \tau)}{T}\right)$$

Where K=1.3695e^{-8} is a conversion factor from rad/s to mm/s. The Γ of the four inner lines are broadened by a fitted factor, A (lines 2 and 5) or B (lines 3 and 4), which is multiplied by W_{relax}. Although the Wickman method is not a model of the physical reason for the sextet collapse, it provides a simple phenomenological fitting method to simulate the collapse of the sextet structure.

In order to test the benefits of using a dynamic relaxation model, we used a version of the Blume-Tjon model[14], as implemented in the SYNCMoss software [15]. This is a simplified stochastic model of the flipping of the magnetic moment, which describes the physical reason for dynamic relaxation in Mössbauer spectra. The line width, Γ, could not provide an accurate replacement for the distribution of the hyperfine field when fitted, and was therefore fixed. This represents a typical baseline of broadening for static spectra. Numerical integration of a gaussian distribution of B_{hf}, ε and **δ** could not be implemented due to the computational intensity of that method. Therefore, we implemented gaussian smearing of the six transition peaks using the distribution of B_{hf} and factors 0.161, 0.093 and 0.026 mm/s per T for the outer, middle and inner lines, respectively. These factors are the magnetic field sensitivity of each of the nuclear energy transitions that produce the peaks observed in a sextet, and the numerical factors are derived from the fundamental physical constants of ^{57}Fe and the respective nuclear state transitions involved in Zeeman splitting. All peaks were smeared by the sum of squares of the line-specific widths, σ(**δ**) and (3 σ(**δ**)).

For each of the models, the fitted parameters for energy-domain spectra (velocity against absorption) were coupled to the parameters used for spectra at all other temperatures. Therefore, these spectral parameters were defined by their values at B_{hf}-field saturation (0 K) and a definition of their relationship with temperature.

To begin, δ was related to θ_D [16]:

$$\delta = \delta_{sat} - \left(\frac{9k_B}{16Mc}\right)(1 + 8\left(\frac{T}{\theta_D}\right)^4 \int_0^{\frac{\theta_D}{T}} \frac{x^3}{e^x - 1} dx)$$

Where the mass of the nucleus, M = 9.6732796×10^{-23}. The first term, δ_{sat}, is the temperature-independent isomer shift, and the second term relates to the second order doppler shift.

In the fitting models presented here, QS(T) was assumed to be constant for model simplicity, although it can also be tied to lattice vibration for more precise fitting of this parameter [17]. The ε for the sextet was calculated from the QS [18]:

$$\varepsilon = (3cos^2(\theta) - 1)\left(\frac{QS}{4}\right)$$

Where θ is the fitted angle of the nuclear spin. The σ(QS) and σ(ε) were fitted separately and not linked to one another mathematically.

The dependence of the hyperfine field, B_{hf}, on measurement temperature was defined by the Brillouin function[19]:

$$H = B_{sat}\left(\frac{6}{5}\coth\left(\frac{6x}{5}\right) - \frac{1}{5}\coth\left(\frac{x}{5}\right)\right)$$

Where the constants assume that the total angular momentum of the material, J = 5/2, which is consistent with the electron configuration of Fe(III). The magnetic field strength at each temperature, x, is defined by the Bean-Rodbell model [20]:

$$x = \frac{15}{7}\frac{T_B}{T}\frac{B_{hf}}{B_{sat}}\left(1 + \nu\left(\frac{B_{hf}}{B_{sat}}\right)^2\right)$$

Where ν is a fitted scaling factor for the gradient of the curve. In the protocols using static relaxation models, the value of ν may be considered an empirical value that is tied to the particle properties of the material, distorting the original physical meaning of the parameter. Since B_{hf} is both the solution of the Brillouin function and an input for the Bean-Rodbell function, it must be solved recursively, for which we used the 'fixed point' function in the 'SciPy' Python library [10] with 3000 iterations and tolerance of 1×10^{-3}.

The area of all sextets and doublets was normalised by the Lamb-Mössbauer factor, also known as the f-factor, $f = e^{W_T}$, where W_T is the Debye-Waller factor:[21]

$$W_T = \left(\frac{6E_R}{k_B}\right)\left(\frac{1}{\theta_D}\right)\left(\frac{1}{4} + \left(\frac{T}{\theta_D}\right)^2 \int_0^{\frac{\theta_D}{T}} \frac{x}{e^x - 1}dx\right)$$

The constant $6E_R/k_B$ is approximated as 136.5.

Each fitting procedure used the Nead-Melder optimisation method implemented with the 'Scipy optimize' package [10] with a maximum of 2500 iterations.

Artificial intelligence tools (Goole's Gemini and Microsoft Copilot; various versions available throughout 2025 and 2026) were used in protected subscription environments to draft sections of code and to aid in the process of debugging code. All AI generated code has been checked by human proofreading against primary sources for authenticity and accuracy.

S1.4. Additional supporting analysis: X-ray diffraction

The 2L-Fh and 6L-Fh were measured by X-ray diffraction (XRD) to check the identity and purity of the minerals. Dried solid samples were resuspended in ethanol and dropped onto polished Si wafers (711 cut, Sil'tronix Silicon Technologies, France). The ethanol was allowed to evaporate leaving the ferrihydrite in pace. Samples were analysed in Bragg–Brentano geometry using Cu Kα radiation ($\lambda_{K\alpha1}$ = 1.540562 Å, $\lambda_{K\alpha2}$ = 1.544398 Å, 40 kV, 40 mA) and a high-resolution energy-dispersive 1D detector (D8 Advance, Bruker, USA; LYNXEYE detector). Measurements were taken between 10° and 70° 2θ with a step size of 0.02° 2θ and measurement time of 4 s/step.

S2. Supporting Analytical Results

S2.1. X-ray diffraction

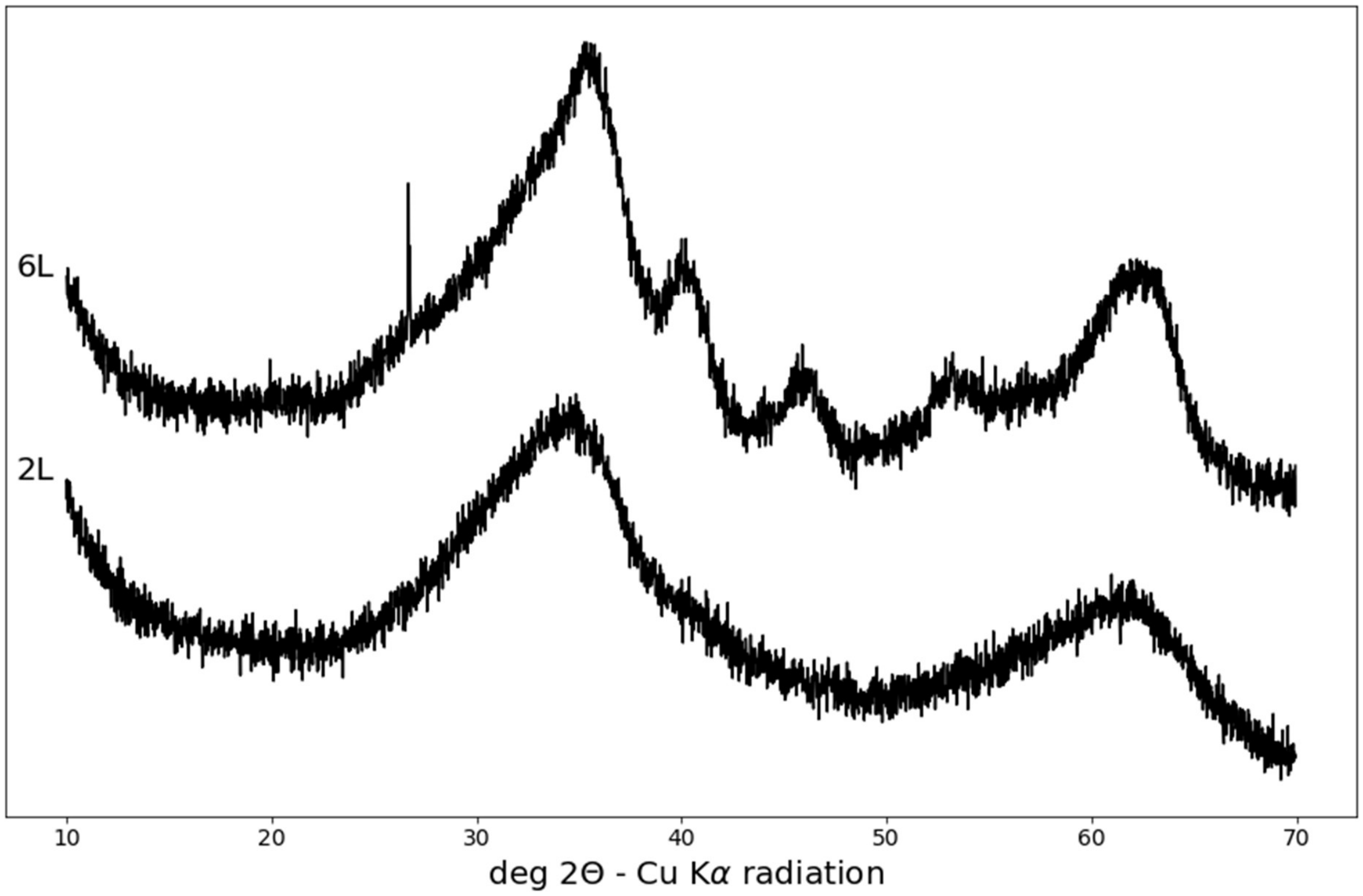

Figure S1: *XRD patterns of 2-line (2L, bottom) and 6-line (6-L, top) ferrihydrite. Diffraction patterns of the coprecipitated ferrihydrite do not clearly show the ferrihydrite signal as the ferrihydrite enrichment was small and the signal is dominated by the well-defined peaks of the crystalline minerals that are naturally present in the soil. Small quartz peaks are visible in the 6L-Fh XRD pattern. Method is outlined in Section S1.4.*

S2.2. Mössbauer Collection Software

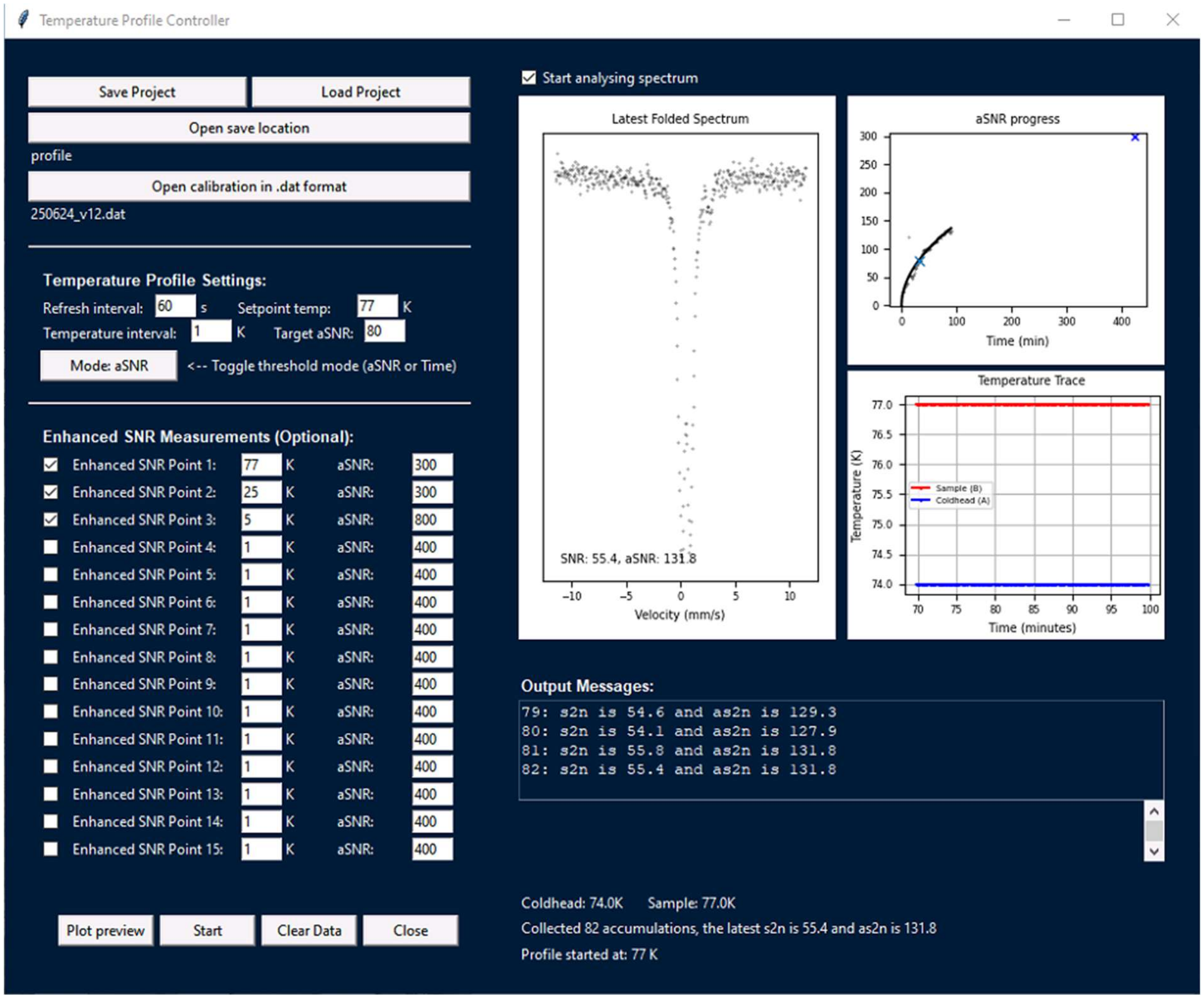

Figure S2: *Screenshot of the GUI which was created with the Tkinter python package [22] and used to automate the collection of Mössbauer spectra.*

S2.3. Mössbauer spectral results and data

Table S1: Fitting parameters of high SNR spectra of 2L-Fh, fitted at each temperature independently, and displayed in Figure 1. The reported hyperfine parameters are centre shift (δ), quadrupole splitting (QS), quadrupole shift (ε) average hyperfine field strength (B_{hf}) and average B_{hf} adjusted to remove negative parts of the distributions (<|B_{hf}|>). Each of QS, ε and B_{hf} are fitted with a Gaussian distribution with standard deviation σ. The goodness-of-fit is indicated by the reduced chi squared (χ^2) and the normalised root mean square error (NRMSE).

Temp	Phase	Rel. spec. area	δ	QS or ε	σQS or σε	B_{hf}	σB_{hf}	<|B_{hf}|>	σ<|B_{hf}|>	chi2	NRMSE
5.0 K	Sextet	1	0.47	-0.01	0.21	48.79	1.99	48.79	1.99	0.000182	4.53
15.0 K	Sextet	1	0.47	-0.01	0.23	47.72	2.15	47.72	2.15	0.000208	5.01
25.0 K	Sextet	1	0.47	-0.01	0.29	45.64	2.53	45.64	2.53	0.000281	6.26
35.0 K	Sextet	1	0.47	-0.01	0.39	42.15	3.26	42.15	3.26	0.000389	8.03
45.0 K	Sextet	1	0.47	-0.01	0.41	35.26	5.52	35.26	5.52	0.00037	8.47
55.0 K	Doublet	0.22	0.45	2.00	1.80	N/A	N/A	N/A	N/A	3.1E-05	2.30
55.0 K	Sextet	0.78	0.46	-0.01	0.56	28.84	5.46	28.84	5.46	3.1E-05	2.30
65.0 K	Doublet	0.40	0.45	1.71	1.68	N/A	N/A	N/A	N/A	1.01E-05	0.93
65.0 K	Sextet	0.60	0.48	0.00	-0.08	17.85	7.90	17.92	7.75	1.01E-05	0.93
75.0 K	Doublet 1	0.41	0.46	0.93	0.69	N/A	N/A	N/A	N/A	1.29E-05	0.94
75.0 K	Doublet 2	0.30	0.44	1.45	1.28	N/A	N/A	N/A	N/A	1.29E-05	0.94
75.0 K	Sextet	0.29	0.47	0.00	0.54	0.80	10.15	8.12	6.13	1.29E-05	0.94
85.0 K	Doublet	1	0.46	1.03	0.90	N/A	N/A	N/A	N/A	9.12E-05	2.49
95.0 K	Doublet	1	0.45	1.01	0.89	N/A	N/A	N/A	N/A	8.17E-05	2.32
105.0 K	Doublet	1	0.45	1.00	0.87	N/A	N/A	N/A	N/A	8.04E-05	2.29
115.0 K	Doublet	1	0.45	1.00	0.85	N/A	N/A	N/A	N/A	6.99E-05	2.18
125.0 K	Doublet 1	0.66	0.44	0.91	0.67	N/A	N/A	N/A	N/A	2.33E-05	1.34
125.0 K	Doublet 2	0.34	0.48	1.70	1.67	N/A	N/A	N/A	N/A	2.33E-05	1.34
135.0 K	Doublet	1	0.44	0.99	0.83	N/A	N/A	N/A	N/A	8.04E-05	2.47

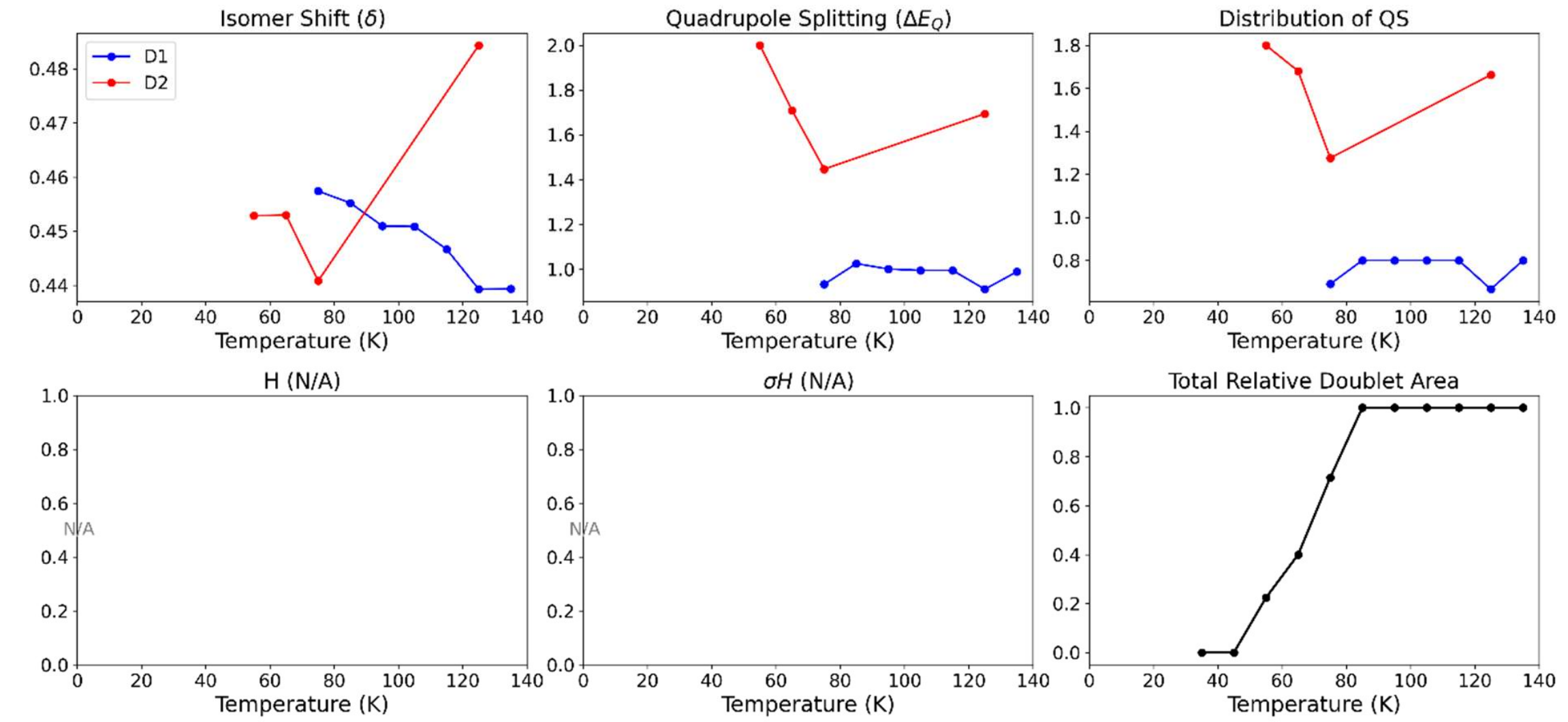

Figure S3: *Parameters of the fitted doublets in the low temperature-resolution profile of 2L-Fh. The relevant data are presented in Table S1.*

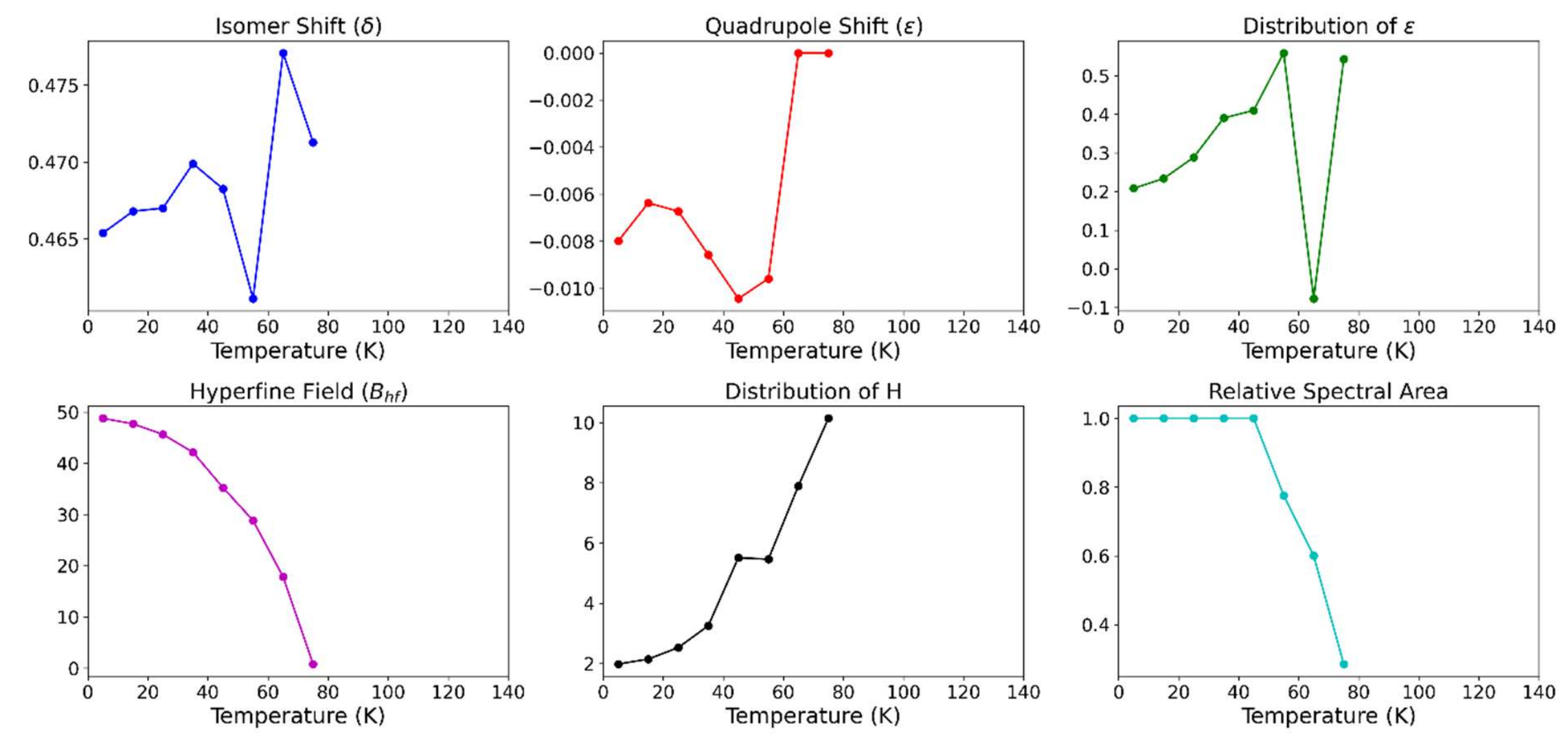

Figure S4: *Parameters of the fitted sextets in the low temperature-resolution profile of 2L-Fh. The relevant data are presented in Table S1.*

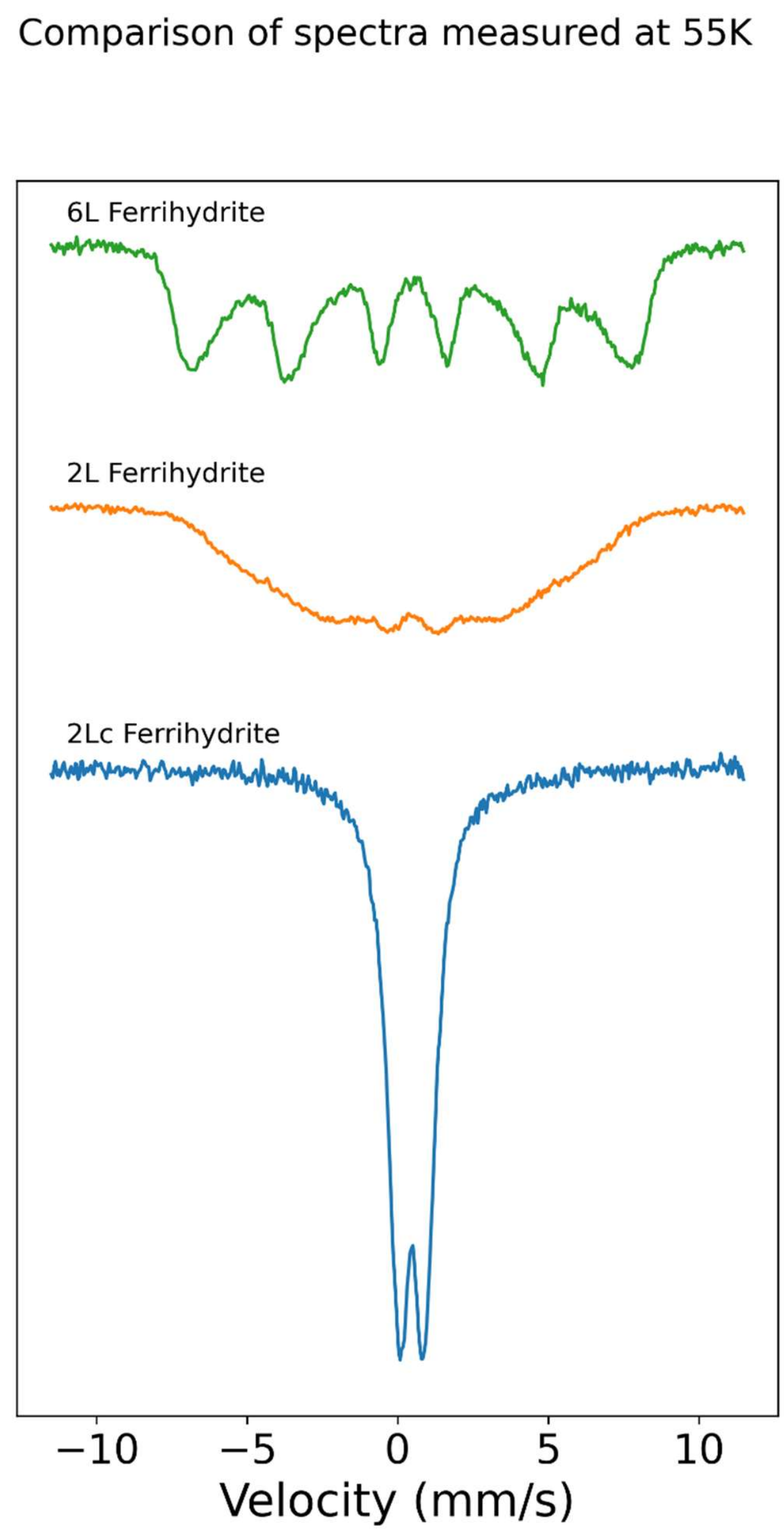

Figure S5: *The relative T_B of phases can be compared at a well-chosen temperature. Here, the Mössbauer spectra collected at 55 K show that the T_B of 2Lc-Fh is lowest, the T_B of 6L-Fh is highest, and T_B of 2L-Fh falls in between.*

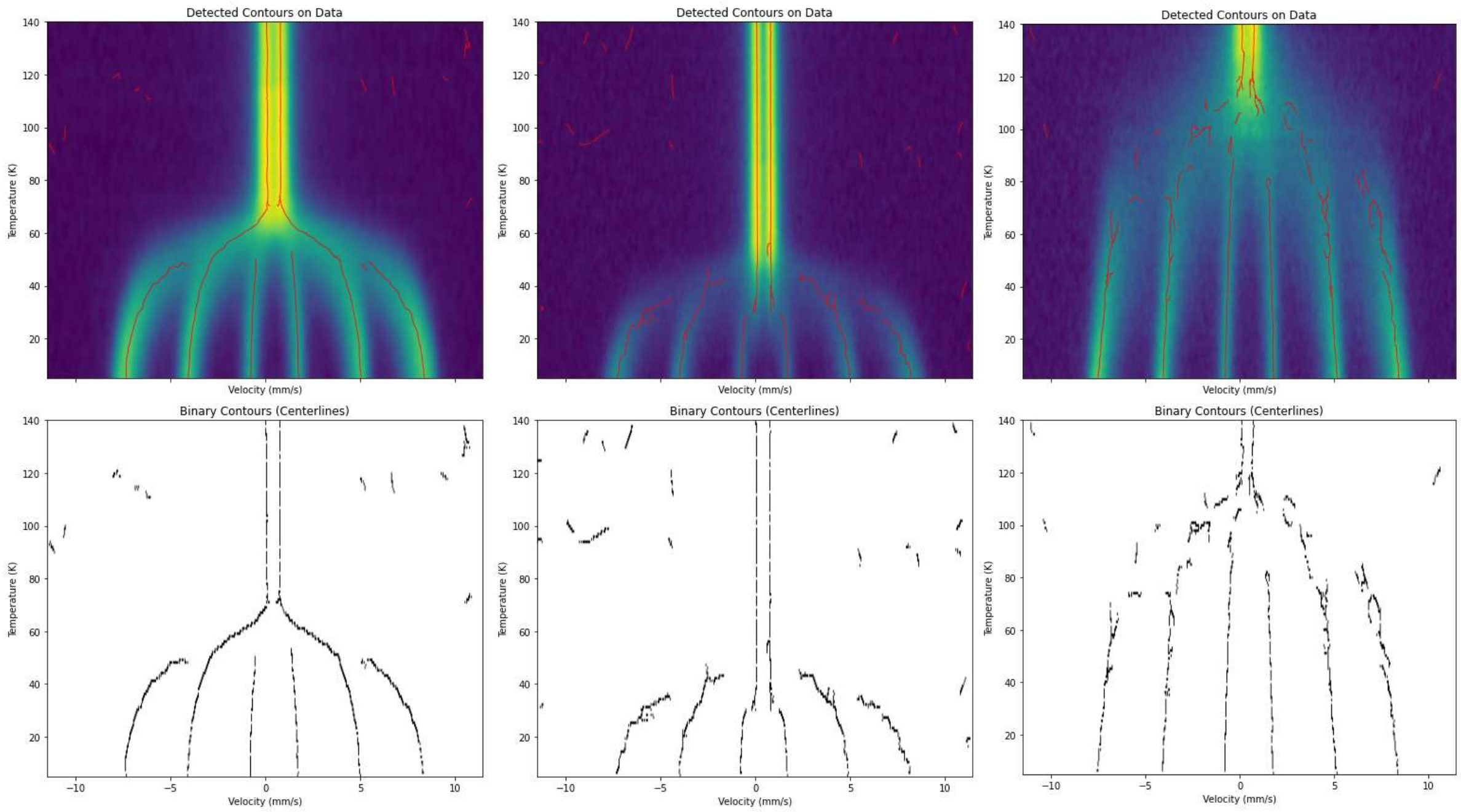

Figure S6: *Image processing techniques can be used to highlight the characteristic absorption patterns in the 2D data. The top images overlay the detected valley lines on the data for 2L-Fh (left) 2Lc-Fh (middle) and 6L-Fh (right). The bottom images show the same detected lines without the data. In this example, the data was pre-smoothed with a gaussian kernel with 1SD of 1 pixel in each dimension.*

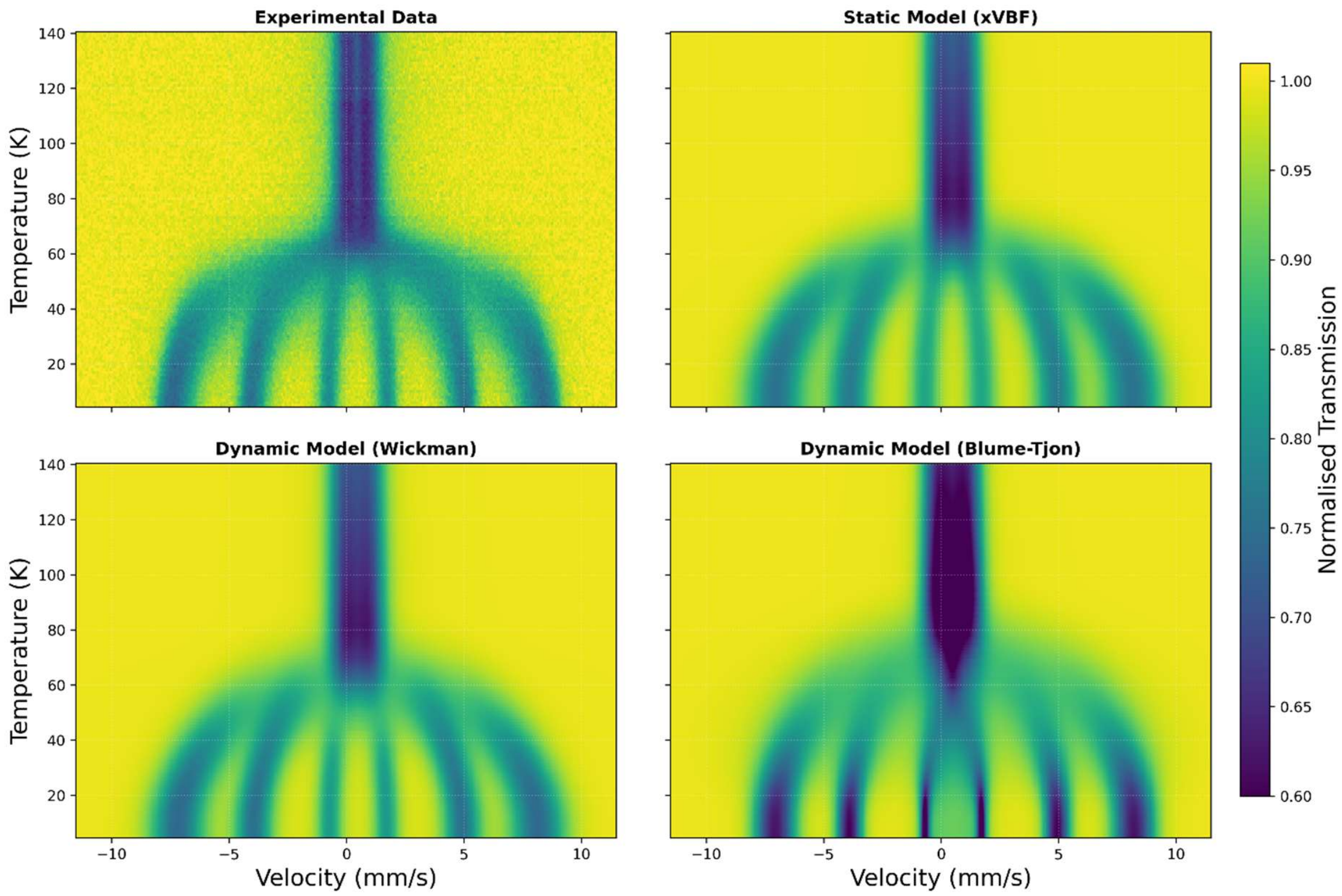

Figure S7: *Comparison of 2L-Fh data plotted in matrix format and the fitted spectra using three different fitting protocols as described in the text.*

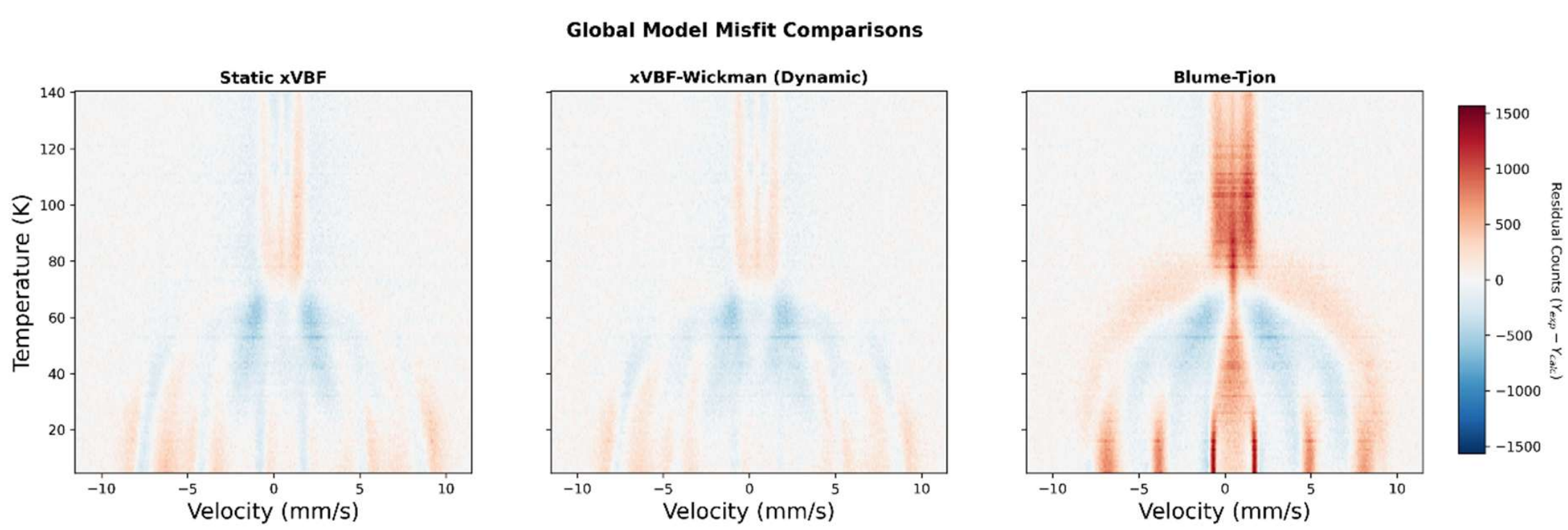

Figure S8: *Comparison of the misfit between the 2L-Fh data and the fitted profile using the three different fitting protocols.*

S3. Additional explanation regarding the effect of the distribution of T_B on spectral line shape.

A novel aspect of the fitting regimes is that they account for the effect of the distribution of the T_B on the line shape of spectra at individual temperatures. Figure S7 illustrates the effect of the distribution of T_B on the asymmetry of sextet peaks. A gaussian distribution of T_B is defined in Panel A, which is then reduced to a series of example particles in Panel B. The example particles are distributed according to the density of the gaussian distribution of T_B. All of the example particles have a saturated hyperfine field at T = 0 K, B_{hf} = 0 T at their individual T_B, and B_{hf} at temperatures in between that are calculated by the Brillouin function, with the magnetisation determined by the Bean-Rodbell model (Section S1.3). If we measure a spectrum at any given temperature, we see the combined B_{hf} of all particles at once. Near T = 0 K, the hyperfine fields of all particles are all close to saturation, and the width of the resulting spectral peak is symmetrical because it is mostly defined by the Voigt curve (σ=0.5 T, Γ=0.135 mm/s) that is used as the basis of the model for each example particle. However, at higher temperatures, the width of the peak is also defined by the variation in the progression of the B_{hf} of each particle towards 0 T at its own T_B, which tends to pull the distribution of the hyperfine field towards the centre. These peak shapes are shown in Panel C, and each curve is colour coded to match the dotted lines in panel B. The significance of this relationship is its application to Mössbauer spectral fitting. This relationship partially explains the shape of static broadening of collapsed sextets. It provides important context for the application of static models to the fitting of high-resolution temperature profiles, as demonstrated in this article. It should also influence how Mössbauer spectroscopists approach the fitting of single-temperature spectra, and how the asymmetry of sextets is explained.

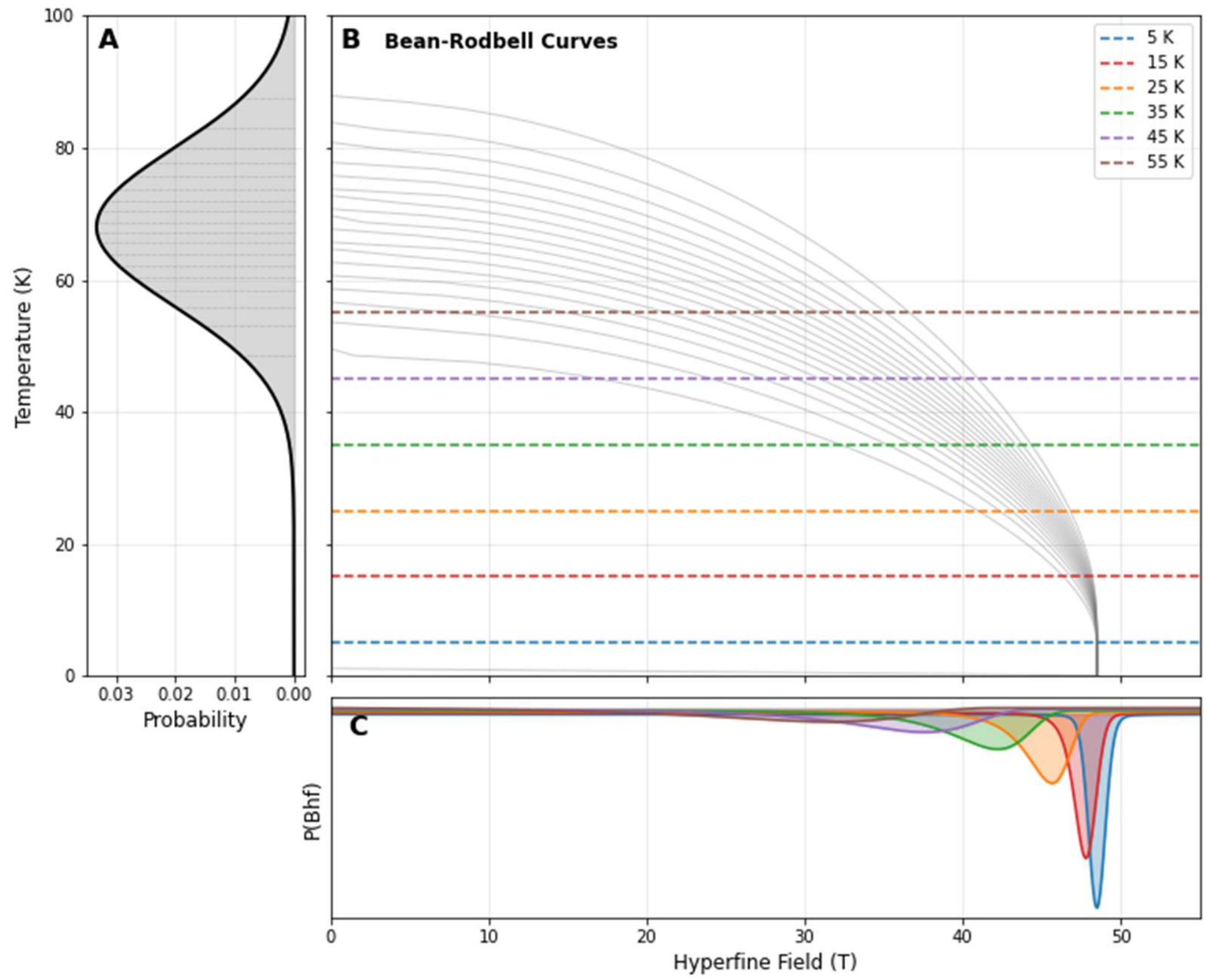

Figure S9: The distribution of the hyperfine field of a Mössbauer spectrum is dependent on the distribution of the T_B in a sample and the relationship between the magnetisation and temperature. **Panel A:** A hypothetical gaussian distribution (σ = 15 K) in particles around $\overline{T_B}$= 68 K. **Panel B:** The calculated Brillouin function for 20 hypothetical particles in the sample with saturated B_{hf}=48.5 T and ν = 0.3. **Panel C:** The resulting hyperfine field distributions at a range of temperatures between 0 and T_B. The colours of the hyperfine distribution curves correspond to the colours of the horizontal lines in Panel B.

S4. Data Availability

All spectral data presented in this paper with the code for the automated collection of the data is available on Github in the 'high-temp-res-mossbauer-profiles' repository on GitHub (https://github.com/Andrew-R-C-Grigg/high-temp-res-mossbauer-profiles) and via the Zenodo repository [7].

S5. References

[1] Carta, D., Casula, M. F., Corrias, A., Falqui, A., Navarra, G., Pinna, G., "Structural and magnetic characterization of synthetic ferrihydrite nanoparticles," *Materials Chemistry and Physics* 113, no. 1 (2009), https://doi.org/https://doi.org/10.1016/j.matchemphys.2008.07.122.

[2] Schwertmann, U., Cornell, R. M. 2000, *Iron Oxides in the Laboratory: Preparation and Characterization, 2e*. Weinheim: Wiley-VCH Verlag GmbH.

[3] Hofmann, A., Pelletier, M., Michot, L., Stradner, A., Schurtenberger, P., Kretzschmar, R., "Characterization of the pores in hydrous ferric oxide aggregates formed by freezing and thawing," *J. Colloid Interface Sci.* 271, no. 1 (2004).

[4] McMahon, M., Hermann, M., Open Source Contributors 2025, "pywinauto - Windows GUI Automation with Python." Github.

[5] a)López, J. H., Restrepo, J., Barrero, C. A., Tobón, J. E., Ramírez, L. F., Jaramillo, J., "Autonomous sample switcher for Mössbauer spectroscopy," *Hyperfine Interactions* 238, no. 1 (2017), https://doi.org/10.1007/s10751-017-1424-8; b)Gütlich, P., Bill, E., Trautwein, A. X. 2011, "Experimental." in *Mössbauer Spectroscopy and Transition Metal Chemistry: Fundamentals and Applications*. edited by Gütlich, P., Bill, E., Trautwein, A. X., Berlin, Heidelberg: Springer Berlin Heidelberg. ISBN 978-3-540-88428-6.

[6] Lake shore Cryogenics 2024, "lakeshore: A package to connect to and interact with Lake Shore instruments." PyPI repository, URL: https://pypi.org/project/lakeshore/.

[7] Grigg, A. R. C., Byrne, J. M., Kretzschmar, R. 2026, "Automated Acquisition, Processing & Fitting of High Temperature-Resolution Mössbauer Spectroscopy (Nussbaum)." v1.0.0 Ed.: Zenodo. DOI: 10.5281/zenodo.21366740.

[8] Lin, G., "A multi-scale ridge detector for identifying curvilinear structures in images," *URL: https://pypi.org/project/ridge-detector/#ridge_detector-0.1.2.tar.gz* (2024).

[9] Lagarec, K., Rancourt, D. G., "Extended Voigt-based analytic lineshape method for determining N-dimensional correlated hyperfine parameter distributions in Mössbauer spectroscopy," *Nucl. Instrum. Methods in Phys. Res. B* 129, no. 2 (1997).

[10] Virtanen, P., Gommers, R., Oliphant, T. E., et al., "SciPy 1.0: Fundamental algorithms for scientific computing in Python," *Nat. Methods* 17, no. 3 (2020).

[11] Byrne, J. M., "MinSight – a new concept for fitting and interpreting Mössbauer spectroscopy data," *Interactions* 246, no. 111 (2025), https://doi.org/https://doi.org/10.1007/s10751-025-02331-7.

[12] Wickman, H. H., Klein, M. P., Shirley, D. A., "Paramagnetic Hyperfine Structure and Relaxation Effects in Mössbauer Spectra: Fe^{57} in Ferrichrome *A*," *Phys. Rev.* 152, no. 1 (1966), https://doi.org/10.1103/PhysRev.152.345.

[13] Mørup, S., Topsøe, H., "Mössbauer studies of thermal excitations in magnetically ordered microcrystals," *App. Phys.* 11, no. 1 (1976), https://doi.org/10.1007/BF00895017.

[14] Blume, M., Tjon, J. A., "Mössbauer Spectra in a Fluctuating Environment," *Phys. Rev.* 165, no. 2 (1968), https://doi.org/10.1103/PhysRev.165.446.

[15] Yaroslavtsev, S., "SYNCmoss software package for fitting Mossbauer spectra measured with a synchrotron Mossbauer source," *J. Sync. Radiat.* 30, no. 3 (2023), https://doi.org/doi:10.1107/S1600577523001686.

[16] Gütlich, P., Bill, E., Trautwein, A. X. 2011, "Hyperfine Interactions." in *Mössbauer Spectroscopy and Transition Metal Chemistry: Fundamentals and Applications*. edited by Gütlich, P., Bill, E., Trautwein, A. X., Berlin, Heidelberg: Springer Berlin Heidelberg. ISBN 978-3-540-88428-6.

[17] Verma, H. C., Rao, G. N., "Quadrupole interactions at ^{57}Fe in Te metal," *Physical Review B* 22, no. 7 (1980), https://doi.org/10.1103/PhysRevB.22.3152.

[18] Greenwood, N. N., Gibb, T. C. 1971, "Hyperfine Interactions." in *Mössbauer Spectroscopy*. edited by Greenwood, N. N., Gibb, T. C., Dordrecht: Springer Netherlands. ISBN 978-94-009-5697-1.

[19] Cullity, B. D., Graham, C. D. 2009, *Instroduction to Magnetic Materials, 2nd ed.* Hoboken, N. J.: Wiley.

[20] Bean, C. P., Rodbell, D. S., "Magnetic Disorder as a First-Order Phase Transformation," *Phys. Rev.* 126, no. 1 (1962), https://doi.org/10.1103/PhysRev.126.104.

[21] Gütlich, P., Bill, E., Trautwein, A. X. 2011, "Basic Physical Concepts." in *Mössbauer Spectroscopy and Transition Metal Chemistry: Fundamentals and Applications*. edited by Gütlich, P., Bill, E., Trautwein, A. X., Berlin, Heidelberg: Springer Berlin Heidelberg. ISBN 978-3-540-88428-6.
[22] Lundh, F., "An introduction to tkinter," *URL: www. pythonware. com/library/tkinter/introduction/index. htm* (1999).
